# Incidence of adverse events in an integrated US healthcare system: a retrospective observational study of 82,784 surgical hospitalizations

**DOI:** 10.1186/1754-9493-8-23

**Published:** 2014-05-27

**Authors:** Muhammad F Zeeshan, Allard E Dembe, Eric E Seiber, Bo Lu

**Affiliations:** 1 Department of Community Health Sciences, Peshawar Medical College, Peshawar, Pakistan; 2 Division of Health Services Management & Policy, Center for Health Outcomes, Policy and Evaluation Studies, The Ohio State University College of Public Health, 283 Cunz Hall, 1841 Neil Avenue, Columbus, OH 43210, USA; 3 College of Public Health Division of Health Services, Management and Policy, The Ohio State University, 1841 Neil Avenue, Columbus, Ohio 43210, USA; 4 College of Public Health, Division of Biostatistics, The Ohio State University, 1841 Neil Avenue, Columbus, Ohio 43210, USA

**Keywords:** Adverse event, Patient safety, Medical error, Surgery, Rates, Severity, Complications

## Abstract

**Background:**

Many health care facilities have developed electronic reporting systems for identifying and reporting adverse events (AEs), so that measures can be taken to improve patient safety. Although several studies have examined AEs in surgical settings, there has not previously been a systematic assessment of the variations in adverse event rates among different types of surgery, nor an identification of the particular types of AEs that are most common within each surgical category. Additionally, this study will identify the AE severity level associated with each of the AE category types.

**Methods:**

This retrospective observational study was conducted at three Midwestern hospitals that are part of a large integrated healthcare system. Data from 2006 through 2009 were analyzed to determine the rates of reported adverse events (per 1,000 hospitalizations involving a surgical procedure) for 96 categories of surgery as classified according to the ICD-9-CM procedural coding system. Univariate and bivariate summary statistics were compiled for AEs by type, severity, and patient age.

**Results:**

During the four-year study period, there was a total of 82,784 distinct hospitalizations involving at least one surgical procedure at these three hospitals. At least one adverse event was reported at 5,368 (6.5%) of those hospitalizations. The mean rate of AEs among all surgical procedure groups was 82.8 AEs per 1,000 hospitalizations. Adverse event rates varied widely among surgical categories with a high of 556.7 AEs per 1,000 hospitalizations for operations on the heart and pericardium. The most common type of adverse event involved care management, followed by medication events and events related to invasive procedures.

**Conclusions:**

Detecting variations in AEs among surgical categories can be useful for surgeons and for hospital quality assurance personnel. Documenting the specific AE incidence rates among the most common types of surgical categories, and determining AE severity and age distributions within surgical categories will enable officials to better identify specific patient safety needs and develop appropriately targeted interventions for improvement.

## Background

Improving the quality of care by minimizing surgical complications and adverse events (AEs) is an important goal for surgeons. To this end, several national initiatives have been launched in the U.S. to enhance the quality of surgical care and the avoidance of surgical errors [1-3]. Improving surgical quality requires data systems for reporting and categorizing problems that occur. Many hospitals and integrated health systems nationally now have an electronic event reporting system (ERS) to identify and analyze AEs, so that appropriate quality assurance measures can be undertaken [4,5].

Several studies have been conducted using an ERS to describe the extent and type of AEs reported in hospital settings. For example, Milch et al. [6] analyzed 92,547 AEs from 26 acute-care hospitals. Their study found that 33% of the AEs were related to medication errors, 15% involved laboratory problems, 13% were falls, 13% were administrative mistakes, and the remaining 19% were miscellaneous non-medication errors [6]. A similar study at an academic medical center in Missouri found that 26% of events were medication-related, 11% were related to therapeutic interventions, 9% were falls, and there was a large variety of miscellaneous events [7]. Paradis’ et al. [8] ERS-based study at three hospitals in Oregon found that 38% of events were medication errors, 39% were the result of treatment procedures, and 9% were related to falls [8].

In the surgical field, it has been estimated that surgery related AEs occur in 1.9% to 3.6% of all hospitalizations, representing 46% to 65% of all AEs [9-11]. A few studies of AE reporting have been conducted for specific surgical subspecialties, including dermatologic, cardiac, and orthopedic surgery [12-15]. This study aims to provide more comprehensive information than has previously been available about the rates of AE occurrence across major surgical categories, and the types of AEs that are associated with specific varieties of surgery. Stratified analyses of the data will describe patterns of AE severity and the distribution of AEs by patient age range.

Several national initiatives, such as the American College of Surgeons National Surgical Quality Improvement Program (NSQIP), have been established to capture standardized information about the type and rate of surgically-related adverse events. Such systems are generally based on collecting data utilizing uniform event reporting protocols. While standardized reporting procedures promotes uniformity and benchmarking, the use of a facility-based or hospital system specific event reporting system has the potential benefit of enabling more detailed and comprehensive information than would be available by the use of national standardized AE reporting systems alone. There may be a significant advantage, for example, to capture surgical event information during the pre-operative and post-operative phases of the hospitalization to provide information on contextual events (e.g., falls) related to the surgical episode.

The accuracy of reporting adverse events is heavily dependent on the completeness, precision, and motivation of the individuals collecting and recording that information. Barriers to accurate reporting of AEs include the reluctance of some medical providers (particularly physicians) to report AEs, lack of time needed to report events because of workload pressures, availability and complexity of the ERS systems, and fear about the repercussions of reporting errors in practice. There may also be reporting bias with regards to the type of events reported (e.g., “near miss” events). For these reasons, it is likely that not all AEs will be reported, and that estimates of AE rates may therefore represent an underestimate of true event prevalence.

To address these potential problems, the hospital system involved in this study takes a variety of actions to achieve complete and accurate reporting. The system’s quality assurance department provides extensive training to clinicians and staff in use of the ERS system, actively encourages reporting and the development of a culture of reporting within the hospital system, maintains a well-staffed quality improvement unit that records all events and near misses, investigates reported events, double checks ERS system reports against information contained in electronic clinical medical records, and incentivizes hospital personnel to file AE reports without fear of reprisal.

## Methods

This retrospective records-based study was conducted at three Midwestern hospitals in the United States that are part of a large integrated healthcare system. All three hospitals conduct inpatient surgeries. Study subjects included all adult (at least 18 years old) patients receiving an inpatient surgical procedure who were admitted and discharged between January 1, 2006 through December 31, 2009. Demographic characteristics of the study subjects are summarized in Table 1.

**Table 1:** Study population demographic characteristics (n = 82,784)

**Age (mean)**	55.7 years
**Age (% distribution)**	
18-24 years	4.1%
25-44 years	21.6%
45-64 years	44.2%
65-84 years	27.5%
85+ years	2.6%
**Gender**	
Female	47.5%
Male	52.5%
**Race**	
White	77.4%
African American	18.8%
Asian	0.6%
Other	1.4%
Not reported	1.8%
**Ethnicity**	
Hispanic	0.8%
Non-Hispanic	85.7%
Not reported	13.5%
**Insurance type**	
Commercial	27.9%
Medicare	29.0%
Medicaid	27.8%
Other	39.1%
**Year of discharge**	
2006	17.6%
2007	25.0%
2008	28.5%
2009	27.9%

The three hospitals shared a common ERS, which is administered by the quality control department of the healthcare system. Patients’ de-identified records were available from the system’s information warehouse, which consolidates electronic medical records of patients across the three hospitals. Records were excluded from this study if a subject’s electronic medical record did not contain an indication of the patient’s primary surgical procedure. Electronic records were coded to protect patient identities. The study protocol was approved by the hospital system’s institutional review board.

Hospitals within this system use an ERS that was initially developed in 2004, primarily for quality improvement purposes. All clinical staff within the system receive extensive training on event identification and reporting. All reports of AEs are entered into the ERS system including detailed information about the AE. For this study, we adopted a relatively broad definition of what constitutes an *adverse event,* capturing events both with and without resulting harm to the patient, and recorded events as well as reported “near misses”. Reported adverse events in this analysis thus encompass both the concepts of “adverse event” and “medical error” as used in the U.S, Institutes of Medicine 1999 report, *To Err is Human*” [16].

### Study variables

Data concerning patient’s age, gender, race, ethnicity, insurance status, and admission/discharge dates were collected from the hospital system’s information warehouse along with clinical variables such as duration of hospitalization, primary ICD-9-CM code, and the primary surgical procedure performed during the hospitalization. Related surgical groups were identified using the first two digits of the ICD-9-CM classification system for surgical, diagnostic, and therapeutic procedures. There were a total of 100 surgical groups identified for the analysis. A surgical hospitalization was defined as an episode of care consisting of an admission and discharge and that involved an ICD-9-CM surgical procedure. Four of the 100 surgical groups (ICD-9-CM codes 72, 73, 90 and 94) did not have any surgical procedures performed during the four-year study period, and therefore were excluded from the analysis.

Data obtained from the hospital system’s ERS included the number of reported AEs for a patient during a surgical hospitalization, and the type of AE based on coding schemes inherent within the ERS. Additionally, the ERS system contained a seven-level severity score for each reported AE. We recoded that severity index into three levels of low severity (levels 0–1), moderate severity (levels 2–3), and high severity (levels 4–6). The severity levels used within the system’s ERS are described in Table 2.

**Table 2:** Adverse event severity levels

Level 0	Did not reach patient
Level 1	No change in patient outcome
Level 2	Increased monitoring required; therapy changed/held/discontinued
Level 3	Additional labs or diagnostic tests were ordered; vital signs changed
Level 4	Reason for admission; prolonged length of stay
Level 5	Transfer to ICU; monitored bed required; invasive procedure required
Level 6	Patient death
Recoding	levels 0–1 = mild severity
	levels 2–3 = moderate severity
	levels 4–6 = substantial severity

The ERS coding consisted of nine categories of AEs (care management, transfusion, clinical support services, falls, invasive procedures, medications, equipment/devices, patient/visitor behavior, and a miscellaneous “other” category), each subdivided into additional more specific codes. For example, AEs in the “care management” category were divided into the following subcategories: omission of care, delay in care, lack of documentation, delay in treatment, and care coordination problems.

### Statistical analysis

Descriptive statistics were compiled summarizing the frequency of surgical hospitalizations, AEs, the number of surgical hospitalizations in which AEs occurred, and the number of surgical procedures, stratified by each of the 96 surgical groups. For brevity of presentation, we only reported the top 15 surgical categories in each analysis. The rate of AEs per 1,000 surgical hospitalizations and AEs per 1,000 surgical procedures were calculated for each of those ICD-9-CM groupings. The percent distribution of types of AEs within each surgical group was also determined, as was the distribution of AE types by severity levels of AEs and of reported AEs by age group. A t-test for Pearson’s correlation coefficient was performed to determine the extent of correlation between the volume of surgeries performed and the reported AE rates within surgical groups. Statistical analyses were performed using Stata statistical software, version 12 (StataCorp, LP, College Station, Texas, USA).

## Results

During the four-year study period, there was a total of 82,784 surgical hospitalizations involving 87,119 surgical procedures; an average of 1.05 procedures per surgical hospitalization (Table 3). There were 5,368 distinct surgical hospitalizations in which at least one AE was reported, an average rate of 1 AE reported for every 15.4 surgical hospitalizations (6.5%). There were 6,856 AEs overall, an average of 1.28 AEs per surgical hospitalization, since one hospitalization can involve multiple AEs.

**Table 3:** Ranking of top 15 surgical categories, by the rate of AEs per 1,000 hospitalizations (Stays)*

**Procedure code (ICD-9-CM)**	**Surgical procedure category**	**Rate of AEs per 1,000 stays**	**Number of AEs**	**Total stays**	**Stays with AEs**	**Total procedures**
37	Operations on heart and pericardium	556.7	329	591	204	750
35	Operations on valves and septa of heart	446.1	426	955	296	1,112
50	Operations on liver	418.7	85	203	53	256
31	Other operations on larynx and trachea	371.7	278	748	162	955
34	Operations on chest wall, pleura, mediastinum, and diaphragm	350.6	122	348	87	421
1	Incision and excision of skull, brain, and cerebral meninges	331.8	210	633	156	730
43	Incision and excision of stomach	331.5	61	184	46	217
52	Operations on pancreas	320.1	89	278	66	328
41	Operations on bone marrow and spleen	270.7	36	133	25	157
32	Excision of lung and bronchus	266.7	72	270	55	309
45	Incision, excision, and anastomosis of intestine	259.5	362	1,395	266	1,645
46	Other operations on intestine	248.3	110	443	94	511
36	Operations on vessels of heart	242.6	214	882	178	986
74	Cesarean section and removal of fetus	228.1	13	57	11	60
84	Other procedures of musculoskeletal system	215.9	220	1,019	169	1,250
Total for top 15 categories		322.8	2,627	8,139	1,868	9,687
Total for all 96 categories		82.8	6,856	82,784	5,368	87,119

*In this table, surgical hospitalizations are abbreviated as “stays” for brevity.

The mean rate of AEs among all surgical procedure groups was 82.8 AEs per 1,000 surgical hospitalizations. But there was considerable variation in the rate of AEs among ICD-9-CM procedural groups (S.D. = 115.8). For example, the 15 surgical categories with the highest rates of AEs averaged 322.8 AEs per 1,000 surgical hospitalizations, compared to a rate of 5.9 AEs per 1,000 surgical hospitalizations among the 15 surgical categories having the lowest AE rates per 1,000 hospitalizations. Surgical categories with the highest rates of AEs included operations on the heart and pericardium (556.7 AEs per 1,000 hospitalizations), valves and septa of the heart (446.1), liver (418.7), and larynx and trachea (371.7) (see Table 3).

Table 4 specifies AE rates among the 15 most commonly performed types of surgery, which included repair and plastic operations on joint structures (ICD-CM code 81), incisions of skin and subcutaneous tissue (code 86), incision and excision of joint structures (code 80), and operations on lens (code 13). Those 15 surgical categories had a comparatively low AE rate (average of 60.6 AEs per 1,000 surgical hospitalizations), leading us to speculate that surgical categories that are performed frequently might generally have lower rates of AEs than types of surgeries that are performed comparatively less often (e.g., because of the greater experience a hospital has in performing such surgeries). However, subsequent analyses performed to test that assumption found a relatively low correlation across all 96 surgical categories (r = −0.056) and no statistically significant relationship (p =0.59) between surgical volume in a particular category and AE rates.

**Table 4:** Ranking of top 15 surgical procedure categories, by the volume of procedures

**Procedure code (ICD-9-CM)**	**Surgical procedure category**	**Total procedures**	**Number of AEs**	Total stays*	**Stays with AEs**	**Rate of AEs per 1,000 stays**
81	Repair and plastic operations on joint structures	5,840	407	5,694	356	71.5
86	Incision of skin and subcutaneous tissue	5,542	436	5,064	342	86.1
80	Incision and excision of joint structures	4,904	102	4,823	86	21.1
13	Operations on lens	4,789	18	4,782	18	3.8
39	Other operations on vessels	4,654	455	4,274	358	106.5
79	Reduction of fracture and dislocation	3,020	213	2,744	183	77.6
83	Operations on muscle, fascia, and bursa, except hand	2,824	123	2,705	100	45.5
53	Repair of hernia	2,644	85	2,604	68	32.6
77	Incision, excision, and division of other bones	2,599	101	2,516	90	40.1
4	Operations on cranial and peripheral nerves	2,392	49	2,374	44	20.6
51	Operations on gallbladder and biliary tract	2,256	131	2,223	109	58.9
55	Operations on kidney	2,024	245	1,839	195	133.2
38	Incision, excision, and occlusion of vessels	1,963	258	1,806	201	142.9
68	Other incision and excision of uterus	1,873	116	1,861	105	62.3
44	Other operations on stomach	1,767	108	1,701	88	63.5
Total for top 15 categories		49,091	2,847	47,010	2,343	60.6
Total for all 96 categories		87,119	6,856	82,784	5,368	82.8

*In this table, surgical hospitalizations are abbreviated as “stays” for brevity.

The distribution of specific types of reported AEs also varied considerably across surgical categories (Table 5). Overall, the most common type of surgical AE involved care management (e.g., delay in care, omission of care, lack of documentation), accounting for 20.8% of all AEs, followed by medication-related AEs (19.2%). Among surgical categories with the highest AE rates, medication-related AEs were most common, followed by AEs related to care management. Table 6 summarizes the distribution of types of AEs among the most frequently performed surgical categories. The analysis of severity levels by type of AE indicated that the highest levels of severity were found in AEs related to invasive and surgical procedures (Table 7). Results of the analysis by age groups indicated that AEs involving care management issues increased in relationship to increasing patient age. Also, there was a consistent trend observed between advancing patient age and the occurrence of adverse events involving falls (Table 8).

**Table 5:** Percent distribution of types of AEs by surgical procedure categories, in top 15 rank order by AE rate/1,000 stays*

**Procedure code (ICD-9-CM)**	**Surgical procedure category**	**Rate of AEs per 1,000 stays**	**% Care management**	**% Transfusions**	**% Clinical support services**	**% Falls**	**% Medications**	**% Invasive procedures**	**% Other**
37	Operations on heart and pericardium	556.7	18.0	14.9	13.1	5.8	16.8	23.8	7.6
35	Operations on valves and septa of heart	446.1	16.4	14.1	12.9	5.4	22.5	21.8	6.8
50	Operations on liver	418.7	14.1	17.6	3.5	11.8	20.0	18.8	14.1
31	Other operations on larynx and trachea	371.7	24.5	10.4	18.7	4.3	21.6	10.1	10.4
34	Operations on chest wall, pleura, mediastinum, & diaphragm	350.6	18.0	12.3	13.1	4.9	28.7	11.5	11.5
1	Incision and excision of skull, brain, and cerebral meninges	331.8	11.9	9.0	11.0	15.2	22.9	20.0	10.0
43	Incision and excision of stomach	331.5	23.0	16.4	9.8	8.2	24.6	13.1	4.9
52	Operations on pancreas	320.1	13.5	5.6	15.7	9.0	21.3	30.3	4.5
41	Operations on bone marrow and spleen	270.7	8.6	20.0	5.7	8.6	14.3	28.6	14.3
32	Excision of lung and bronchus	266.7	22.5	8.5	16.9	4.2	22.5	15.5	9.9
45	Incision, excision, and anastomosis of intestine	259.5	20.8	13.0	15.0	9.1	21.6	13.6	6.9
46	Other operations on intestine	248.3	30.9	10.9	12.7	3.6	22.7	12.7	6.4
36	Operations on vessels of heart	242.6	11.2	19.6	13.1	5.1	18.2	23.8	8.9
74	Cesarean section and removal of fetus	228.1	23.1	0.0	7.7	0.0	15.4	23.1	30.8
84	Other procedures of musculoskeletal system	215.9	27.8	8.3	11.1	16.2	17.1	10.6	8.8
Overall for all surgical groups		82.8	20.8	11.3	12.5	8.5	19.2	17.8	9.9

*In this table, surgical hospitalizations are abbreviated as “stays” for brevity.

**Table 6:** Percent distribution of types of AEs among surgical procedure categories, in Top 15 rank order by volume of procedures

**Procedure codes (ICD-9-CM)**	**Surgical procedure categories**	**Total procedures**	**% Care management**	**% Transfusions**	**% Clinical support services**	**% Falls**	**% Medications**	**% Invasive procedures**	**% Other**
81	Repair and plastic operations on joint structures	5,840	35.0	7.8	9.2	8.9	15.3	13.1	10.8
86	Incision of skin and subcutaneous tissue	5,542	27.9	10.9	14.2	12.7	19.0	6.6	8.6
80	Incision and excision of joint structures	4,904	31.2	5.2	7.8	11.7	27.3	9.1	7.8
13	Operations on lens	4,789	0.0	0.0	0.0	0.0	66.7	33.3	0.0
39	Other operations on vessels	4,654	16.6	15.2	12.7	10.4	17.1	20.3	7.8
79	Reduction of fracture and dislocation	3,020	19.9	15.4	5.0	11.9	14.4	18.4	14.9
83	Operations on muscle, fascia, and bursa, except hand	2,824	29.4	12.7	13.7	8.8	18.6	10.8	5.9
53	Repair of hernia	2,644	21.3	9.3	12.0	13.3	24.0	10.7	9.3
77	Incision, excision, and division of other bones	2,599	20.2	8.3	11.9	8.3	15.5	14.3	21.4
4	Operations on cranial and peripheral nerves	2,392	17.6	5.9	14.7	8.8	17.6	26.5	8.8
51	Operations on gallbladder and biliary tract	2,256	22.4	13.6	12.0	4.0	24.8	16.8	6.4
55	Operations on kidney	2,024	11.9	12.7	12.7	11.1	20.1	20.9	10.7
38	Incision, excision, and occlusion of vessels	1,963	20.6	8.6	11.5	8.2	18.5	21.4	11.1
68	Other incision and excision of uterus	1,873	19.0	13.0	7.0	4.0	18.0	24.0	15.0
44	Other operations on stomach	1,767	24.1	10.2	18.5	4.6	19.4	16.7	6.5
Overall for all surgical groups		87,119	20.8	11.3	12.5	8.5	19.2	17.8	9.9

**Table 7:** AE types by age groups, percent distribution (%)

**Age groups (yrs)**	**Care management**	**Transfusion**	**Clinical support services**	**Falls**	**Medications**	**Invasive-procedural**	**Other**	**Total RAEs**
18-24	19.51	16.03	10.45	4.53	15.33	20.21	13.94	287
25-44	19.73	9.37	13.81	8.17	18.89	18.60	11.42	1,419
45-64	20.78	10.41	12.20	8.49	20.20	17.24	10.70	3,075
65-84	21.09	13.01	12.75	9.23	18.78	17.94	7.19	1,906
85 and above	27.81	14.20	8.88	11.24	14.20	15.98	7.69	169
Overall (%, sum)	20.77	11.25	12.53	8.53	19.18	17.81	9.93	6,856

**Table 8:** Percent (%) distribution of severity levels, by type of AE

**AE severity level**	**Care management**	**Transfusions**	**Clinical support services**	**Falls**	**Medications**	**Invasive/procedural**	**Others**	**Total AEs (number)**
Mild severity	45.6	65.2	67.9	19.8	44.1	35.7	59.3	3,272
Moderate severity	44.9	31.3	28.1	74.7	51.4	49.9	36.4	3,091
Substantial severity	9.5	3.5	4.1	5.5	4.5	14.4	4.3	493
Total number of AEs	1,424	771	859	585	1,315	1,221	681	6,856

## Discussion

There has been increasing interest in measuring and reporting the occurrence of adverse events in the United States during the 2000s and 2010s. Many hospitals and health care systems routinely collect this information and use it in quality assurance efforts. While there have been some studies directed at determining the rates of adverse events for particular kinds of surgeries, we believe that this current study provides one of the most comprehensive assessments to date, spanning such areas as determining the rate of AEs among leading ICD-9-CM surgical codes, identifying the surgical categories having the greatest volume of AEs, determining the distribution of AEs by specific AE types, specifying the distribution of AE types by age group, and providing information about the types of surgically-related AEs in which the highest severity AEs occur.

Our analysis has found that there is very wide variation in AE rates across surgical categories, with a rate of 322.8 AEs per 1,000 hospitalizations for the top 15 categories compared to a mean of 82.8 AEs per 1,000 hospitalizations for the set of all 96 available 2-digit ICD-9-CM surgical categories. On average, at least 1 AE was reported for every 15.4 surgical hospitalizations (6.5% of hospitalizations). The variations in AEs could reflect differences in underlying surgical complexity and/or indicate clinical safety-related issues related to a particular surgical category.

The overall rate of 82.8 AEs per 1,000 surgical hospitalizations is somewhat higher than the rate cited in other published studies. The broad definition we adopted of what constituted an “adverse event”, including near misses and events without patient harm, may have influenced that observed rate. Additionally, this health system maintains a sophisticated AE reporting system, and its clinicians and quality assurance personnel are trained to be particularly thorough and comprehensive in the collection, investigation, and reporting of AE data. Results from this facility, therefore, may not necessarily be generalizable to other health systems.

Our experience in utilizing an ERS to derive comparative statistics of AE rates across surgical categories demonstrates the potential usefulness of these methods to help support quality assurance and improvement objectives within a hospital system. For example, in this particular application, knowing that surgical procedures of the heart, pericardium, valves and septa of the heart, larynx and trachea, and liver have resulted in the highest rates of AEs will facilitate the development of special interventions, intensified investigation, and dialogue with affected surgeons and support personnel to develop appropriate responses and countermeasures.

Similarly, our analysis found that orthopedic surgeries, especially joint repair and joint tissue incision and excision are numerically the most common surgical categories in which AEs occur. Identifying and documenting those trends helps to focus attention and resources at that area so that effective improvement programs can be developed. For example, a high volume of adverse events occurring in a particular orthopedic surgery unit could spur the development of enhanced communications techniques or acquisition of new information technology.

The further linking of AEs rates within surgical categories to specific varieties of AEs (e.g., medication-related, falls, or delays in treatment) further refines the ability of quality improvement personnel and surgeons to identify strategies for mitigating AE risks. In this regard, it was interesting to see the extent to which there was a strong relationship between increasing patient age and increasing incidence of AEs in some AE categories (e.g.,, care management and falls) but not in other AE categories (e.g., medications and transfusions). It was also particularly interesting to observe an unexpected trend between increasing patient age and a *lower* frequency of AEs related to invasive procedures. Additional investigation will be needed to fully understand the reasons underlying those patterns of AE occurrence.

Based on preliminary findings of seemingly low AE rates among many of the surgical categories having a high volume of procedures, we speculated that a high volume of a particular category of surgical procedures might be related to lower AE rates. This speculation was grounded in our knowledge of other studies that have shown an association between surgical volume (a particular surgeon’s volume, or volume within a particular hospital) and clinical outcomes in a variety of settings [17,18]. We therefore attempted to ascertain whether the volume of procedures within our data set showed a relationship of lower AE risks with increased volume of procedures among surgical categories. No statistically significant associations were detected based upon our preliminary examination using simple correlation analysis techniques. Further inquiry to examine potential relationships between AE rates and surgical procedure volume in particular categories might be warranted.

One of the advantages of the ERS used in this study was the inclusion of a scale for categorizing the severity of adverse events. This potentially allows for identification of the AE types that are most likely to result in severe AEs. The severity scale used by the healthcare system in this study was designed and used for quality assurance purposes only within this system. Not every health care institution has a similar process for grading the severity of a particular AE. Including a severity scale is beneficial because it potentially enables more precise evaluation and response, along with more efficient use of time and resources. There have been some efforts at the national level to create uniform AE scaling techniques, such as the Common Terminology Criteria for Adverse Events severity scale created by the National Cancer Institute [19]. However, in general, there is still a need to better incorporate uniform severity scales into AE reporting systems.

This study involved three hospitals that are part of a single integrated healthcare system. A limitation of this study is that the results from this system might not be representative of other hospitals or health care settings. Because many health care systems do not publicly share data about adverse event occurrence, it is often challenging to determine whether data about adverse events is collected and interpreted in a sufficiently similar way to make the findings generalizable.

It is thus difficult to compare our results against other institutions because there are few uniform standards or coding systems for event reporting that are used across different hospital systems. To enhance patient safety reporting in the future, hospital systems may need to work cooperatively to develop standardized approaches for event reporting that facilitate benchmarking and trending using common data, while protecting the confidential nature of event data. At the national levels a variety of initiatives are underway to aggregate adverse events data to derive more globally applicable information.

However, many national adverse event reporting initiatives -- such as those undertaken by the National Quality Forum, the Agency for Healthcare Research and Quality, the Joint Commission, and the National Surgical Quality Improvement Program -- rely on a relatively small set of common indicators and thus lack the richness, detail, and variety that can often be obtained through use of a customized hospital-based ERS of the type utilized in this study [20-22]. Surgeons need to pay attention to the data that can be obtained by the use of facility or system-specific event reporting systems. For instance, unlike the event reporting system used at the system featured in this study, the NSQIP surgical event reporting initiative does not as yet contain a severity-grading protocol applicable to diverse surgical procedures. The ideal reporting system would feature uniform AE coding and data collection processes that would enable benchmarking with other healthcare systems, but that also would be detailed and comprehensive enough to meet the specific quality assurance needs of a particular institution.

## Conclusions

The extreme variations in AE incidence rates may reflect patient safety issues in some clinical units and/or underlying differences in surgical complexity. Hospital systems need to identify units with the highest rates of AE occurrence and AE severity so that remediation and response efforts can be appropriately targeted. Further sub-analysis to determine patterns in the specific type of AE relative to the type of surgical category can be helpful in further refining quality assurance and patient safety efforts. While individual facilities can benefit by having a detailed and customized approach to AE data collection and analysis, it may also be helpful to adopt uniform data concerning AE occurrence among health facilities as a way of benchmarking results with other institutions.

## Competing interests

The authors declare that they have no competing interests.

## Authors’ contributions

MZ was the primary developer of the study design, conducted most of the statistical analyses, and was the primary author of the manuscript. AD participated in the study design and authoring of the manuscript. ES provided advice on the study’s methodology, data integrity, and helped revise and refine the final manuscript. BL provided assistance of study design and statistical methods, along with review of draft and final manuscripts. All authors read and approved the final manuscript.
